# Correction to “Role of tumor necrosis factor‐α and its receptors in human benign breast lesions and tumors (in situ and infiltrative)”

**DOI:** 10.1111/cas.16145

**Published:** 2024-03-18

García‐Tuñón, I., Ricote, M., Ruiz, A., Fraile, B., Paniagua, R. and Royuela, M. (2006), Role of tumor necrosis factor‐α and its receptors in human benign breast lesions and tumors (in situ and infiltrative). Cancer Science, 97: 1044–1049. https://doi.org/10.1111/j.1349‐7006.2006.00277.x

The Western blot bands in Figure 1 were cropped and pasted into a standard background. This type of image processing does not comply with the current best practice. Yet, at the time of publication, journal policies regarding image processing did not explicitly require a description of the image amendments in the figure caption. The authors confirmed that the cropped bands of each marker were run on the same gel and that all the experimental results and corresponding conclusions mentioned in the paper remain unaffected. The authors apologize for this error. Please find the updated caption of Figure 1 as follows.

**FIGURE 1** Western blot analysis of tumor necrosis factor (TNF)‐α, TNF receptor (TNFR) I and TNFRII after 15% sodium dodecylsulfate–polyacrylamide gel electrophoresis. BL, benign lesions; IC, infiltrating carcinoma; ISC, in situ carcinoma. The bands of each marker were cropped from the same gel and juxtaposed against a standard background.

